# Association between Femoral Artery Flow-Mediated Dilation and Muscle Oxygen Saturation Parameters in Healthy, Young Individuals

**DOI:** 10.3390/jcdd10020063

**Published:** 2023-02-03

**Authors:** Vivian dos Santos Pinheiro, Anna Carolina Faria da Silva Tavares, Mônica Volino-Souza, Gustavo Vieira de Oliveira, Thiago Silveira Alvares

**Affiliations:** 1 Nutrition and Exercise Metabolism Research Group, Multidisciplinary Center, Nutrition Institute, Federal University of Rio de Janeiro, Macaé 27979-000, Brazil; 2 Postgraduate Program in Food Science, Federal University of Rio de Janeiro, Rio de Janeiro 21941-909, Brazil; 3 Postgraduate Program in Bioactive Products and Biosciences, Federal University of Rio de Janeiro, Macaé 27979-000, Brazil; 4 Multidisciplinary Center, Institute of Medical Sciences, Federal University of Rio de Janeiro, Macaé 27979-000, Brazil

**Keywords:** endothelial function, microvascular responsiveness, reactive hyperemia, femoral FMD, anterior tibial oxygenation

## Abstract

Flow-mediated dilation (FMD) and muscle oxygen saturation (StO_2_) are measurements utilized to assess macro- and microvascular function, respectively. Macro- and microvascular dysfunction may occur differently depending on the clinical condition. Since microvascular responsiveness can influence upstream conduit artery hemodynamics, the present study aimed to investigate whether a correlation between FMD and muscle StO_2_ parameters exists. Sixteen healthy, young individuals were enrolled in this study. Femoral artery FMD and tibial anterior muscle StO_2_ were evaluated by ultrasound and near-infrared spectroscopy, respectively. The FMD and muscle StO_2_ parameters were assessed by employing a vascular occlusion test (VOT). The oxygen resaturation rate was determined by calculating the upslope of StO_2_ immediately after occlusion and the magnitude of reperfusion as the difference between the highest and lowest StO_2_ value achieved during the reperfusion phase. The oxygen desaturation rate and the magnitude of desaturation during the VOT were also evaluated. A significant correlation between the FMD and oxygen resaturation rate (r = 0.628; *p* = 0.009), magnitude of reperfusion (r = 0.568; *p* = 0.022), oxygen desaturation rate (r = −0.509; *p* = 0.044), and magnitude of desaturation (r = 0.644; *p* = 0.007) was observed. This study demonstrated a moderate association between the femoral artery FMD and tibial anterior StO_2_ parameters in young individuals.

## 1. Introduction

Macro- and microvascular dysfunction is an abnormal condition involved with atherosclerosis, which contributes to cardiovascular events [1]. In this context, determining an easy, low-cost, and fast method for assessing vascular function is crucial to preventing cardiovascular disease, a main cause of death worldwide [2]. Flow-mediation dilation (FMD) is a non-invasive, well-tolerated, and widely utilized method for assessing endothelial function in conduit arteries (i.e., brachial, femoral, popliteal arteries) in the healthy and clinical population [3,4].

Using an ultrasound, the percentage of changes in the conduit artery diameter from the baseline to post-cuff occlusion peak diameter can be evaluated through a vascular occlusion test (VOT). Changes in the artery diameter after a 5-min occlusion occur, since the blood flow suddenly increases immediately after cuff deflation due to downstream vessel dilation (in the region below the cuff) due to the ischemic insult [5,6]. The increased blood flow following the arterial occlusion (i.e., reactive hyperemia) causes a frictional force (i.e., shear stress) on the artery wall, stimulating nitric oxide (NO) production, the main molecule involved in artery dilation during the FMD response [7]. In this sense, reactive hyperemia is a hemodynamic stimulus determining the FMD response, at least when considering healthy, young individuals [4].

Near-infrared spectroscopy (NIRS) assesses muscle oxygen saturation (StO_2_) since NIRS determines changes in oxygenated and deoxygenated hemoglobin in the microcirculation [8]. During the VOT, the muscle StO_2_ parameters during the cuff occlusion and disocclusion are established as a measurement of the magnitude of tissue ischemia and reperfusion, respectively [9,10]. Given that previous studies have found a positive correlation between the FMD response and the parameters of muscle StO_2_ during reperfusion [5,6], the FMD assessment and NIRS analysis have been combined to better understand the crosstalk between macro- and microvascular responsiveness in many clinical populations [4,6,11].

For example, we have shown that the forearm muscle oxygen resaturation rate (assessed during reperfusion) was significantly associated with the brachial FMD response [6,9]. These findings are important given that the NIRS-derived oxygen resaturation rate could be useful for assessing reactivity hyperemia in microcirculation. Most studies reporting a correlation between the FMD response and muscle oxygen resaturation parameters have been evaluated in the upper limb [4,6]. However, the morphology and function of the vasculature in the upper (brachial artery) and lower limb (femoral artery) vary since the upper and lower limb are exposed to different hemodynamic challenges, such as upright posture and bipedal locomotion [12]. Moreover, arteries in the lower limb appear to be more susceptible to atherosclerosis development [13]. The hemodynamic differences between the lower and upper limbs are related to different blood pressures, the regulation of the vascular tonus by the autonomic nervous system, and composition of arterial layers, all of which could contribute to atherosclerosis development [13]. In this sense, the present study aimed to investigate whether a correlation between the femoral artery FMD response and muscle StO_2_ parameters exists in young individuals’ health. We hypothesized that the femoral FMD significatively correlates with muscle StO_2_ parameters during reperfusion.

## 2. Material and Methods

### 2.1. Participants

Sixteen healthy, physically active, and young individuals (eight males and eight females) were recruited through announcements on the University Campus to participate in this study. The baseline characteristics of the participants are shown in Table 1. Individuals of both sexes, aged between 18 and 40 years, were included in this study. The volunteers with the presence of lower limb injury (bone and joint disease, arthritis, etc.), cardiovascular and metabolic diseases (diabetes mellitus, hypertension, lung disease, HIV, and peripheral artery disease), smoking, or taking any supplement that could affect the vascular (vitamins, antioxidants) and muscular (pre-workout, creatine, caffeine) systems were excluded. All experimental procedures were performed in accordance with the ethical standards of the Declaration of Helsinki and approved by the Institutional Ethics Committee of the Federal University of Rio de Janeiro, Multidisciplinary Center UFRJ-Macaé, Brazil (protocol CAAE 55184922.5.0000.5699), and all participants were fully informed about the procedures and gave their informed consent in writing.

### 2.2. Experimental Protocol

Upon the arrival of all participants to the Laboratory, they laid down on the examination table to have their macro- and microvascular function assessed after a 10 min resting period in an air-conditioned room (23–25 °C). The anthropometric measurements (height, weight, body mass index, and leg skinfold) were obtained immediately after vascular measurements. One day before the Laboratory visit, the participants were recommended to avoid ingesting caffeine and food rich in nutrients (i.e., L-arginine, L-citrulline, nitrate, nitrite, and polyphenols, among others) that may affect the vascular parameters before the laboratory visit. A list containing the foods that should be avoided was provided to the participants before the Laboratory visit. All experimental procedures were performed by the same investigator between 03:00 and 05:00 p.m.

### 2.3. Macro- and Microvascular Measures

The superficial femoral artery flow-mediated dilation (FMD) and tissue oxygen saturation (StO_2_) of the anterior tibialis muscle were evaluated simultaneously through a vascular occlusion test (VOT) (1 min baseline, 5 min of occlusion, and 3 min of reperfusion) using ultrasound and near-infrared spectroscopy (NIRS), respectively.

### 2.4. Flow-Mediated Dilation Assessment

The flow-mediated dilation in the superficial femoral artery was assessed according to guidelines presented elsewhere [14,15]. A two-dimensional ultrasound with color and spectral Doppler (Prosound Alpha 6^®^, Aloka Co., Tokyo, Japan) and a linear transducer with a frequency of 5.0 to 13.0 MHz (model UST-5413, Aloka Co., Tokyo, Japan) were used. The superficial femoral artery diameter was recorded continuously at the proximal one-third of the thigh to obtain a longitudinal image of the superficial femoral artery throughout the FMD procedure, and video recordings were saved for offline analysis. The superficial femoral artery diameters were determined using a wall-tracking software program (Cardiovascular Suite version 4.2.1, Quipu srl, Pisa, Italy). A pneumatic cuff connected to an automatic rapid inflation system (Hokanson E20 AG101, Bellevue, WA, USA) was used for the arterial occlusion. The pneumatic cuff was placed approximately 2 cm below the lower border of the patella. The absolute FMD was calculated as the difference between the peak (3 sec average value) and baseline (1 min average value) artery diameter. The FMD (%) was determined as the percent change from the baseline to peak arterial diameter.

### 2.5. Muscle Oxygen Saturation Assessment

The muscle StO_2_ was assessed using a near-infrared spectroscopy (NIRS) device (PortaMon, Artinis, Medical Systems). The NIRS device was placed on the skin of the tibialis anterior muscle belly, and the pneumatic cuff was placed approximately 2 cm below the lower border of the patella. A baseline assessment was recorded (1 min), and the pneumatic cuff was inflated at 250 mm Hg for 5 min. Afterwards, the pneumatic cuff was deflated, and the muscle StO_2_ was assessed for 3 min during the reperfusion phase. The muscle oxygen desaturation rate (%.s^−1^) was calculated as the downslope of the StO_2_ signal during the occlusion phase. The desaturation magnitude (%) was calculated as the difference between the highest and lowest StO_2_ value during the occlusion period. The muscle oxygen resaturation rate (%.s^−1^) was calculated as the upslope of the StO_2_ signal over a 10 s window following the cuff release. The reperfusion magnitude (%) was calculated as the difference between the highest and lowest StO_2_ value during the reperfusion period. The muscle StO_2_ parameters during the occlusion and reperfusion phases were used to measure the muscle oxygen extraction (ischemia magnitude) and microvascular responsiveness, respectively [10].

### 2.6. Statistical Analysis

To determine the correlation between the femoral artery FMD and muscle StO_2_ parameters, correlation coefficients (Pearson correlation test) were used. Before the statistical correlation, a normal distribution test (Shapiro–Wilk test) and homoscedasticity (by a scatter diagram) were analyzed. The statistical significance level was assumed when *p* < 0.05, and the results were expressed as the means ± standard deviation (SD). All analyses were performed using a commercially available statistical package (IBM SPSS Statistic version 23 for Mac, Chicago, IL, USA). The graph was generated using Prism 8.0 (GraphPad Software, San Diego, CA, USA).

## 3. Results

The normality and homoscedasticity of the data were not violated. The baseline characteristics of the participants are shown in Table 1. Table 2 shows the femoral artery and tibial muscle diameter at baseline and post-occlusion, the FMD, and the of tibial muscle NIRS parameters data of 16 participants.

**Figure 1:**
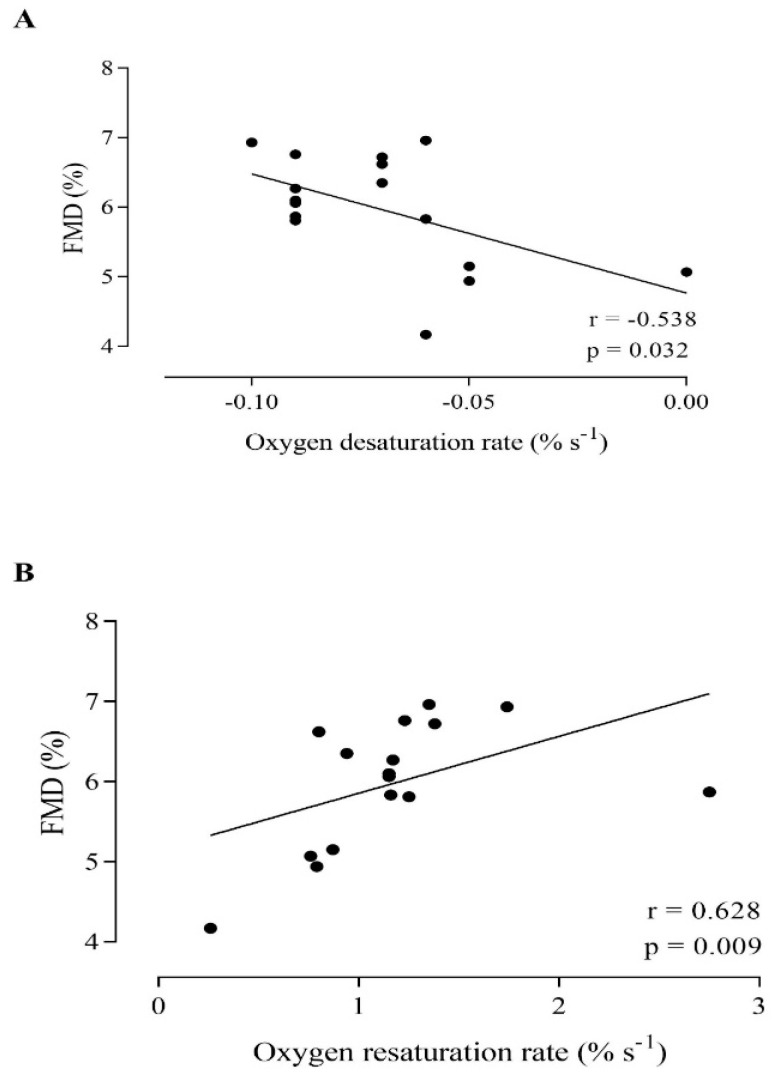
Correlation between the femoral artery flow-mediated dilation (FMD) and tibial anterior desaturation rate (**A**) and resaturation rate (**B**) in healthy, young individuals.

A significant positive correlation between the FMD and the oxygen resaturation rate (r = 0.628; *p* = 0.009) (Figure 1) and the magnitude of reperfusion (r = 0.568; *p* = 0.022) was observed. In relation to the ischemic period, a significant negative correlation between the FMD and the oxygen desaturation rate (r = −0.509; *p* = 0.044) (Figure 1) and a significant positive correlation with the magnitude of desaturation (r = 0.644; *p* = 0.007) were found.

In addition, a significant negative correlation between the oxygen desaturation rate and the magnitude of reperfusion (r = −0.769; *p* < 0.001) and oxygen resaturation rate (r = −0.648; *p* = 0.007) was found. Further, the magnitude of desaturation positively correlates with the magnitude of reperfusion (r = 0.897; *p* = < 0.001). However, no significant correlation between the magnitude of desaturation and the oxygen resaturation rate (r = 0.393; *p* = 0.132) was found.

## 4. Discussion

The present study aimed to investigate whether a correlation between the femoral FMD response and muscle StO_2_ parameters during occlusion and reperfusion periods exists. The findings of this study showed a significant correlation between the FMD and muscle StO_2_ parameters during cuff occlusion (oxygen desaturation rate and magnitude of ischemia) and during reperfusion (oxygen resaturation rate and magnitude of reperfusion).

Upon cuff occlusion, the blood flow to the leg is restricted, and the tissue below the cuff experiences an ischemia period. During ischemia, vasodilator metabolites (i.e., H+ ion, adenosine diphosphate, etc.) dilate arterioles and decrease vascular resistance. Thus, when cuff disocclusion occurs, blood flow suddenly increases in the microvasculature, speeding up the muscle oxygen resaturation rate [4]. The FMD response depends on increased blood flow in the artery to stimulate the vascular endothelium to produce NO, a molecule that induces smooth muscle relaxation surrogating the artery wall (vasodilation). Previous studies have observed a positive correlation between the FMD and muscle oxygen resaturation rate in the arm (brachial artery) [4,6], but not in the thigh (femoral artery), which is a large artery presenting different structures and functions [12].

Soares et al. [16] investigated the association between the brachial artery FMD and forearm muscle oxygen resaturation rate in healthy, young individuals. The authors found a significant positive correlation between the FMD and muscle oxygen resaturation rate, suggesting that reactive hyperemia in the microvasculature seems to affect the FMD response. Moreover, Mclay et al. [5] found a positive correlation between the FMD evaluated in the popliteal artery (leg’s artery) and the muscle StO_2_ resaturation rate assessed in the anterior tibial muscle in healthy, young individuals. In line with our findings, a positive correlation between the femoral artery FMD and oxygen resaturation rate in the anterior tibial muscle was also observed. These findings are interesting given that the femoral artery presents different morphologies and functions compared to the arm’s artery due to the different stressor factors, such as the upright position (which increases the hydrostatic pressure on the artery structure), that the femoral artery undergoes daily [12,16].

The present study also observed a significant correlation between the FMD and muscle StO_2_ parameters during ischemia (oxygen desaturation rate and magnitude of ischemia). From a mechanical perspective, the magnitude of ischemia induced by cuff occlusion depends on the oxidative capacity of muscles experiencing oxygen deprivation. When a muscle extracts a high oxygen level during cuff occlusion, a more robust magnitude of ischemia is generated [10,17]. As a result, by-products from the energetic metabolism can induce microvascular vasodilation, increasing the blood flow velocity in the upstream artery, which results in an increased FMD response. A previous study from our Laboratory failed to show a correlation between the brachial artery FMD and muscle StO_2_ parameters during ischemia [10]. These divergent findings reinforce that the macro and microvascular response observed in the brachial artery should not be extrapolated to the other arteries present in the body’s other segments (i.e., lower limb).

Furthermore, a negative correlation between the oxygen desaturation rate and the magnitude of reperfusion and oxygen resaturation rate was found. It seems that the magnitude of ischemia (i.e., higher muscle oxidative metabolism) may affect the microvascular vasodilation response. A previous study demonstrated a correlation between the magnitude of ischemia (area under the oxygen desaturation curve) and the oxygen desaturation rate in healthy young people and older people at risk for cardiovascular disease [10]. Townsend et al. [18] also found a significant negative correlation between the lowest StO_2_ value reached during cuff occlusion and the oxygen resaturation rate in young patients with insulin resistance. Moreover, Mayeur et al. [19] reported a significant correlation between the extension of muscle oxygen desaturation during ischemia and the oxygen resaturation rate assessed by NIRS in healthy volunteers and septic shock patients.

## 5. Experimental Consideration

A limitation of the present study was not evaluating whether a correlation between the FMD and muscle StO_2_ parameters in the clinical population exists. Such correlation goes beyond the goal of the present study. However, it is important to note that a previous study failed to demonstrate a positive correlation between the FMD and muscle StO_2_ parameters in individuals with cardiovascular risk factors [4], suggesting that abnormal conditions can affect the association between macro- and microvascular responsiveness. Our findings provide information with respect to how reactive hyperemia in the microcirculation (assessed by NIRS technology) could be linked with the FMD response in the upstream conduit artery (femoral artery), which was investigated in the individuals’ thigh. Therefore, studies investigating whether a correlation between the femoral FMD and tibial anterior muscle StO_2_ parameters in the clinical population exists are warranted given that the data from the FMD and StO_2_ parameters in the upper limb should not be extrapolated to the lower limb. Furthermore, we have not evaluated NO metabolites (nitrate, nitrite) in the present study, so a correlation between NO metabolites and the FMD and muscle StO_2_ parameters in young, healthy individuals might have been assessed. However, a previous study has demonstrated a positive correlation between NO and forearm blood flow [20].

## 6. Conclusions

The present study demonstrated a significant correlation between the femoral artery FMD response and tibial anterior muscle StO_2_ parameters assessed during the ischemia and reperfusion periods in healthy, young individuals. These findings are important since they provide information on the impact of reactive hyperemia in the microcirculation of the lower limb on the FMD response assessed in an upstream vessel (femoral artery).

## Figures and Tables

**Table 1:** Baseline characteristics of the participants.

N (male)	16 (8)
Age (years)	27 ± 5
Weight (kg)	69.61 ± 13
Height (m)	1.69 ± 0
Body mass index (kg/m^2^)	24.20 ± 3
Leg skinfold (mm)	7.1 ± 3
Values were expressed as the mean ± standard deviation.	

**Table 2:** Flow-mediated dilation (FMD) and muscle oxygen saturation (StO_2_) parameters throughout a vascular occlusion test in healthy, young individuals.

***Ultrasound-derived parameters***	
Base artery diameter (mm)	5.8 ± 0.83
Peak artery diameter (mm)	6.2 ± 0.91
Flow-mediated dilation (%)	5.9 ± 0.80
***Near-infrared spectroscopy-derived parameters***	
Baseline StO_2_ (%)	71.5 ± 4.06
Desaturation magnitude (%)	19.1 ± 7.83
Oxygen desaturation rate (%.s^−1^)	−0.07 ± 0.02
Reperfusion magnitude (%)	24.3 ± 9.23
Oxygen resaturation rate (%.s^−1^)	1.2 ± 0.54
Values were expressed as the mean ± standard deviation. Abbreviation: StO_2,_ tissue oxygen saturation.	

## Data Availability

The data presented in this study are available on request to the corresponding author. The data are not publicly available due to privacy or ethical restrictions.
